# Risk assessment for prolonged sickness absence due to musculoskeletal disorders: protocol for a prospective cohort study

**DOI:** 10.1186/s12891-020-03354-7

**Published:** 2020-05-25

**Authors:** Anne Therese Tveter, Britt Elin Øiestad, Tarjei Langseth Rysstad, Fiona Aanesen, Alexander Tingulstad, Milada Cvancarova Småstuen, Margreth Grotle

**Affiliations:** 1 Department of Physiotherapy, Faculty of Health Sciences, Oslo Metropolitan University, St.Olavs plass, 0130 Oslo, Norway; 2 National Advisory Unit on Rehabilitation in Rheumatology, Division of Rheumatology and Research, Diakonhjemmet Hospital, Oslo, Norway; 3 Department of Nursing and Health Promotion, Faculty of Health Sciences, Oslo Metropolitan University, Oslo, Norway; 4 Research and Communication Unit for Musculoskeletal Health (FORMI), Oslo University Hospital, Oslo, Norway

## Abstract

**Background:**

Musculoskeletal disorders are the leading cause of sickness absence and disability pension in Norway. There is strong evidence that long-term sickness absence due to musculoskeletal disorders are associated with a reduced probability of return to work (RTW). A way to meet the economic and resource-demanding challenges related to individual follow-up of this group is to identify and treat those individuals with a high risk of prolonged sickness. The overall purposes of this project are 1) to determine the most accurate screening tool to identify people at a high risk of prolonged sickness absence due to an musculoskeletal disorder, and 2) to investigate severity of musculoskeletal health, health-related quality-of-life, health care utilization, and costs across different risk profiles in people on sick leave due to a musculoskeletal disorder.

**Methods:**

People older than 18 years of age on sick leave for at least 4 weeks due to a musculoskeletal disorder will be invited to participate in this prospective observational cohort study conducted within the Norwegian Welfare and Labor Administration (NAV) system in collaboration with OsloMet – Oslo Metropolitan University. The main outcome is sickness absence, obtained from the NAV registry. Data on sickness absence will be retrieved prospectively in the period from study inclusion to 12 months follow-up, and retrospectively 12 months before inclusion in the study. Possible risk factors will be self-reported by the participants at inclusion while health care utilization will be retrieved from registry data. To conduct analyses including 15 to 20 predictor variables, we aim at including 500–600 people on sick leave due to musculoskeletal disorders.

**Discussion:**

This study may provide tools that can be used to identify individuals with high risk of prolonged sickness absence and may thus be important from both a socioeconomic and individual perspective. Further, the study may give valuable insight into identification of sickness absence profiles and the associations between these profiles and musculoskeletal health status, health-related quality of life and costs.

**Trial registration:**

Retrospectively registered in ClinicalTrials.gov (NCT04196634, 27.11.2019).

## Background

Musculoskeletal disorders are a leading cause of years lived with disability worldwide [1, 2] and the prevalence is estimated to increase as the population ages [2, 3]. Importantly, painful musculoskeletal disorders are a common cause of seeking health care [4] and the most common cause of sickness absence and disability pension in Norway [5]. Musculoskeletal disorders accounted for 35–39% of the sickness absence in Norway in 2018 [6], constituting a major health challenge, affecting individuals, their families, employers, health systems and the social care system [3, 7].

Most sickness absence is short-term, however, about one in seven on sick leave is absent for more than 12 weeks [6]. Although this is a rather small proportion of people, they contribute to large costs due to disbursement of benefits, productivity loss and extensive use of health care [5]. There is growing evidence that long-term sickness absence is associated with poorer mental and physical health and well-being [8]. There is strong evidence that long-term sickness absence due to musculoskeletal disorders is associated with a reduced probability of return to work (RTW) when the sick absence exceeds 8 weeks [9–11]. Important modifiable risk factors that negatively affect work participation are symptoms of depression and emotional distress, high pain intensity and disability level, low motivation for RTW, low self-efficacy related to work participation and low work readiness [12, 13]. On the other hand, improved expectations of sickness absence have been associated with a higher probability of RTW [14].

In Norway, the Norwegian Welfare and Labour Administration (NAV) is responsible for the integration and inclusion in working life, preventing withdrawal and sickness absence, and for securing income for those who are unemployed. A governmental goal is to provide different types of interventions to reduce the duration of sickness absence for all people on sick leave, however, this requires enormous resources from the NAV offices. A possible way to meet this challenge is to use risk assessment in order to identify those at high risk of prolonged sickness absence. In a UK study, a stratified care approach, which targeted individual risk factors based on a risk screening tool, succeeded in reducing time off work with 50% among people with non-specific back pain seeking help in primary care [15]. Therefore, a similar approach will be established in Norway within the NAV settings in people on sick-leave due to a musculoskeletal disorder (the MI-NAV project, https://www.muskhealth.com/minav).

In the MI-NAV project, two potential important risk assessment tools will be evaluated; the Örebro Musculoskeletal Pain Screening Questionnaire short form (ÖMPSQ-SF) [16] and the Keele Subgroups for Targeted Treatment (the Keele STarT MSK) tool [17]. It is however, uncertain which instruments are better to predict prolonged sickness absence in people with musculoskeletal disorders.

Finally, the MI-NAV project also includes the recent patient-reported outcome measure for musculoskeletal disorders, the Musculoskeletal Health Questionnaire (MSK-HQ) [18], comprising many of the modifiable risk factors for prolonged sickness absence. However, it has yet to be evaluated outside a clinical setting.

The overall purposes of this project are 1) to determine the most accurate screening tool to identify people at a high risk of prolonged sickness absence due to a musculoskeletal disorder, and 2) to investigate severity of musculoskeletal health, health-related quality-of-life, health care utilization, and costs across different risk profiles in people on sick leave due to musculoskeletal disorders. We will use registry data on sickness absence from 1 year before to 1 year after inclusion in the study. The study will comprise both methodological and predictive sub-studies, of which the specific objectives are outlined below.

Objectives for methodological sub-studies are:

- To translate, cross-culturally adapt and assess measurement properties of the Keele STarT MSK tool and the MSK-HQ tool in people on sick leave due to musculoskeletal disorders.
- To assess the criterion validity of self-reported absenteeism compared to registry data on sickness absence

Objectives for the predictive sub-studies are:

- To compare the predictive ability of the Keele STarT MSK tool and the ÖMPSQ-SF, and other established risk factors for long-term sickness absence (e.g. *symptoms of depression and emotional distress, low motivation for returning to work, low self-efficacy, work expectancies)* for identifying prolonged sickness absence at 6- and 12-months follow-up due to musculoskeletal disorders, including
  - ○ to compare the clinical characteristics of subgroups identified by each tool in people on sick leave due to musculoskeletal pain
  - ○ to assess if the predictive ability is different across different age, sex and socioeconomic groups
  - ○ to determining the optimal cut-off points of the Keele STarT MSK and the ÖMPSQ-SF to identify prolonged sickness absence at 6- and 12-months follow-up
- To develop a prognostic model to predict the risk of prolonged sickness absence at 12-month follow-up in people with musculoskeletal disorders, including
  - ○ To externally validate the prognostic model in other materials in Norway, e.g. the work package 3 of the MI-NAV project and a similar project in Trondheim
- To assess predictors for high costs (productivity loss and health care utilization) at 6- and 12-months follow-up in people on sick leave due to musculoskeletal disorders, including
  - ○ To investigate the use of health care, health-related quality of life, and costs during 12-months of follow-up in people on sick leave due to musculoskeletal disorders
  - ○ To investigate whether sickness absence, use of health care, and costs vary across the specific musculoskeletal disorders (e.g. low back pain, neck, shoulder pain, osteoarthritis) and across different risk profile groups during the 12-months of follow-up
- To explore if clusters of people on sick leave due to musculoskeletal disorders concerning work-related disability, can be identified during a 12-month follow-up (by using latent class analysis), and describe the characteristics of these subgroups with respect to primary and secondary outcomes.

Finally, short reports and several master theses will be conducted using data from the cohort study with different methodological and clinically relevant research questions.

## Methods

### Translation and cultural adaptation of measurements

Prior to the data collection, we translated and culturally adapted the Keele STarT MSK and MSK-HQ following the Beaton guidelines [19]. A bilingual health professional and a bilingual non-medical translator independently performed the translation from English to Norwegian. The questionnaires were then translated back to English by a second pair consisting of a bilingual health professional and a non-medical translator. Possible differences between the back translation and the English version were discussed at a consensus meeting. The translated versions were tested in 42 patients who were either seeking treatment for musculoskeletal conditions at outpatient physiotherapy clinics or who were on sick leave due to a musculoskeletal condition. The patients had the opportunity to either write down comments or answer questions, or both, regarding the understanding of the instructions, questions and the response options, as well as the instrument’s wording. Based on the feedback from the patients, the expert committee discussed the findings and proposed a final version.

### Study design and setting

This is a prospective observational cohort study of people on sick leave due to musculoskeletal disorders. The study is conducted within the Norwegian Welfare and Labour Administration (NAV) system in collaboration with OsloMet – Oslo Metropolitan University. Sickness absence from the NAV registry will be retrieved prospectively in the period from study inclusion to 12 months follow-up, and retrospectively 12 months before inclusion in the study. The present project is a part of a large-scale project (the MI-NAV project) financed by the Research Council of Norway, through the program “Sickness absence, work, and health”.

### Study participants

People older than 18 years of age on sick leave due to a musculoskeletal disorder for at least 4 weeks in Norway will be invited to participate. People on sick leave for other pain conditions or diseases or people not able to understand and write Norwegian or English will be excluded.

### Cohort study recruitment and data collection

**Fig. 1:**
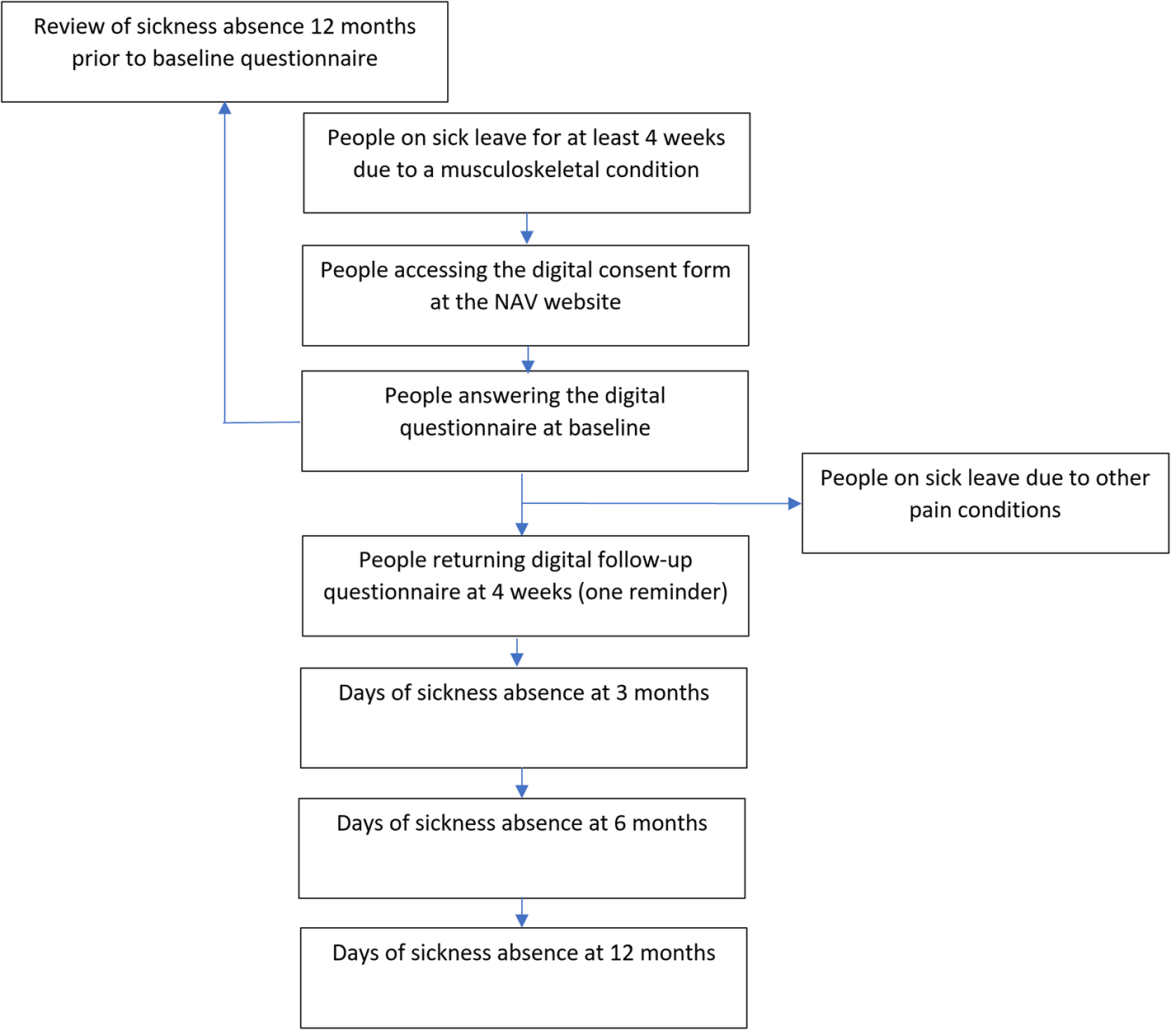
Flow chart of the recruitment procedure. Flow chart of the recruitment procedure for people on sick leave due to a musculoskeletal condition, recruited through the Norwegian Welfare and Labour Administration (NAV)

Eligible participants will be invited to participate electronically through a link on everyone’s individual profile page at the NAV website (Fig. 1). Accessing the link will bring the participants to a consent form. After digitally consenting, the participants are presented with a questionnaire including demographic variables, screening tools for long-term complaints/sickness absence, and questions related to musculoskeletal health, productivity loss and health-related quality of life (Table 1). In addition, the participants will be asked to respond to the electronic questionnaire a second time after 4 weeks. One reminder email will be sent after 3 days to those not answering the questionnaire. Recruitment started in November 2018 and data are still being collected.

**Table 1:** Outcome domains, measures, and timing of data collection

DOMAIN	MEASURE	TIMING OF DATA COLLECTION
**OUTCOME MEASURES**		
Sickness absence	Collected from the NAV registry	12 months before, baseline, 3, 6 and 12 months after baseline
Productivity loss (absenteeism, presenteeism)	iPCQ [20] (summary scores)	Baseline, 4 weeks
Musculoskeletal health	MSK-HQ [18] (summary score)	Baseline, 4 weeks
Health-related quality of life	EQ-5D-5 L [21] (index value)	Baseline, 4 weeks
Use of health care	Collected from public records (NPR, KPR, KUHR)	12 months before, baseline, 3, 6 and 12 months after baseline
**RISK ASSESSMENT MEASURES**		
Bothersomeness	STarT MSK [17] (Q3)	Baseline, 4 weeks
Coping	ÖMPSQ-SF [16] (single item from ÖMPSQ, Q12)	Baseline, 4 weeks
Disability	MSK-HQ [18] (Q4), EQ-5D-5 L [21] (Q2)	Baseline, 4 weeks
Distress	STarT MSK [17] (Q8); ÖMPSQ-SF [16] (Q5,Q6); MSK-HQ [18] (Q11); EQ-5D-5 L [21] (Q5)	Baseline, 4 weeks
Fatigue	MSK-HQ [18] (Q10)	Baseline, 4 weeks
Fear-avoidance beliefs	STarT MSK [17] (Q9); ÖMPSQ-SF [16] (Q9,Q10)	Baseline, 4 weeks
Future disease expectations	STarT MSK [17] (Q6)	
Health literacy	MSK-HQ [18] (Q12)	Baseline, 4 weeks
Independence	MSK-HQ [18] (Q8)	Baseline, 4 weeks
Overall impact	MSK-HQ [18] (Q14)	Baseline, 4 weeks
Pain (management, duration, intensity)	STarT MSK [17] (Q1,Q2,Q5,Q10); ÖMPSQ-SF [16] (Q1,Q2); EQ-5D-5 L [21] (Q4); MSK-HQ [18] (Q1,Q2)	Baseline, 4 weeks
Physical activity	MSK-HQ [18] (Q5,Q15)	Baseline, 4 weeks
Return to work expectancy	ÖMPSQ-SF [16] (Q7,Q8); single item on return to work expectancy [14]	Baseline, 4 weeks
Self-efficacy	MSK-HQ [18] (Q13)	Baseline, 4 weeks
Self-perceived physical function	STarT MSK [17] (Q4); ÖMPSQ-SF [16] (Q3); MSK-HQ [18] (Q3); EQ-5D-5 L [21] (Q1,Q3)	Baseline, 4 weeks
Self-perceived health	STarT MSK [17] (Q7); EQ-5D-5 L [21] (Q6)	Baseline, 4 weeks
Sleep	MSK-HQ [18] (Q9); ÖMPSQ-SF [16] (Q4)	Baseline, 4 weeks
Social activity	MSK-HQ [18] (Q7)	Baseline, 4 weeks
Work conflict	Single question on work conflict (yes/no)	Baseline, 4 weeks
Work information	iPCQ [20]	Baseline, 4 weeks
Work satisfaction	Single item on work satisfaction (Numeric rating scale, 0 = not satisfied, 10 = satisfied); Single question regarding the desire to return to same work position (yes/no)	Baseline, 4 weeks
Workability	Single item from Work Ability Index [22] (Q1, numeric rating scale, 0 = worst, 10 = best); MSK-HQ [18] (Q6)	Baseline, 4 weeks
Change in condition	7-point global rating of change	4 weeks

Abbreviation: *iPCQ* iMTA Productivity Cost Questionnaire, *STarT MSK* Keele Subgroups for Targeted Treatment Musculoskeletal Tool, *ÖMPSQ-SF* Örebro Musculoskeletal Pain Screening Questionnaire short form, *MSK-HQ* Musculoskeletal Health Questionnaire, *EQ-5D-5 L* EuroQol 5 Dimensions, *NPR* Norwegian Patient Registry, *KPR* Municipal Patient and User Registry, *KUHR* Control and Payment of Health Refunds, *Q* Question/item

The study will be conducted according to the Helsinki declaration and participants will sign informed electronic consents before inclusion in the study. Approval has been given by the Norwegian Centre for Research Data (NSD 861249). The project was also reviewed by the Regional Committees for Medical and Health Research Ethics in Norway but was not considered to be medical research and they therefore found it to be beyond the scope of their mandate. Data will be collected electronically, stored and analysed through Services for Sensitive Data (TSD) at the University of Oslo on a secured research server with access only to researchers directly involved in the project.

### Cohort study outcome measures

#### The primary outcome measures

The primary outcomes in the present study will be sickness absence and costs related to health care and sickness absence. Sickness absence will be operationalized in different ways and includes 1) total number of absence days during 6- and 12-month follow-up adjusted for percentage of work and percentage of sickness absence, 2) the time until full sustainable RTW, i.e. at least 4 weeks without relapse during 12-months follow-up, and 3) probability of working (i.e. not receiving medical benefits) each month during 12 months of follow-up, measured as repeated events, and 4) proportion of people with sustainable RTW (at least 4 weeks) at 6 and 12 months. Data on sickness absence will be collected from the NAV registry, containing dates and grading of sickness absence as well as the diagnostic codes related to the absence. The use of health care will be collected from public registries including the Norwegian Patient Registry (NPR), the Municipal Patient and User Registry (KPR), and the Control and Payment of Health Refunds (KUHR). Periods of sickness absence and use of health care will be collected 12 months before inclusion and 6 and 12 months after inclusion in the study (Table 1).

#### The secondary outcome measures

Secondary outcomes will be musculoskeletal health status, health-related quality of life, productivity loss (Table 1). Musculoskeletal health will be measured with *the Musculoskeletal health questionnaire (MSK-HQ)*, which is developed to capture musculoskeletal health status through 15 questions embracing a broad range of musculoskeletal disorders [18]. The first 14 questions are scored on a 0–4-point scale and summed up to a score between 0 and 56 points, with a higher score indicating better musculoskeletal health status. Health-related quality of life will be measured with *the EuroQol 5 Dimensions (EQ-5D-5 L)* [21], which covers five domains: mobility, self-care, activities of daily living, pain/discomfort, and anxiety/depression. The EQ-5D-5 L is scored on a 5-point scale from 1 (no problems) to 5 (extreme problems). Responses can be transformed into an index value ranging from − 0.59 to 1, where − 0.59 represents worst possible state and 1 represents perfect health. The EQ-5D Visual Analogue Scale (VAS) is also included as a measure of health-related quality of life and consists of a single question asking about the respondent’s self-rated health on a vertical 0 to 100 VAS, with 100 indicating best health. Productivity loss will be measured with *The institute of Medical Technology Assessment Productivity Cost Questionnaire (iPCQ)*, which is used to measure and value health-related productivity loss for both paid and unpaid work. The instrument is found to be suitable for measuring absenteeism from paid work and productivity loss related to unpaid labor [20].

### Cohort study risk and demographic variables

The two primary risk assessment tools will be the Keele STarT MSK and ÖMPSQ-SF. *The Keele STarT MSK* tool is a newly developed refined version of the Keele STarT Back Screening tool [23], aimed at identifying a broader range of patients with musculoskeletal disorders at risk of developing long-term pain or disability [17, 24]. The Keele STarT MSK consists of 10 items and the scores are summarized to a 0–12 score, with risk groups being categorized as follows: 0–4 = low risk; 5–8 = medium risk; 9–12 = high risk [17]. *The ÖMPSQ-SF* is a screening tool developed to identify patients at risk of developing work disability due to back pain [16]. The short version contains 10 questions summed up to a score between 0 and 100 [16], with the higher score indicating higher risk [25]. The following demographic variables will be assessed at baseline: sex, age (years), education level (primary/secondary school, high school, higher education up to 4 years, higher education 4 years or more), and diagnosis (ICD-10 Diagnosis code L). Other potential risk factors are presented in Table 1.

### Sample size estimation

Previous studies show that 30–40% of people with musculoskeletal disorders have not RTW after 3 to 12 months [26, 27]. In order to conduct analyses including 15 to 20 predictor variables, we aim at including 500–600 people on sick leave due to musculoskeletal disorders. As the main outcomes are collected through registries, we do not expect any dropouts.

### Statistical analyses

#### Methodological analyses

Measurement properties of the translated questionnaires will be evaluated based on the COnsensus-based Standards for the selection of health Measurement INstruments (COSMIN) guidelines [28, 29]. Construct validity of the Keele STarT MSK and the MSK-HQ will be assessed by testing a priori hypotheses about the relationship with the same and other constructs. For acceptable construct validity, 75% of the hypotheses need to be confirmed. Reliability will be assessed with intraclass correlation coefficient (ICC) using two-way random, average agreement, and smallest detectable change (SDC_95%_) [28]. Criterion validity of self-reported productivity loss (by iPCQ) compared to registered sickness absence will be assessed by Cohen’s unweighted Kappa for dichotomous variables of the iPCQ and by ICC for the index score of absenteeism. According to COSMIN, acceptable level of ICC is > 0.70 [28]. The Kappa values are according to Altman judged as follows: poor (0 to 0.2), fair (0.21 to 0.40), moderate (0.41 to 0.60), good (0.61 to 0.80) and very good (0.81 to 1.00) [30].

#### Predictive analyses

The predictive ability of the Keele STarT MSK, the ÖMPSQ-SF and other established risk factors for long-term sickness absence for detecting people at risk of prolonged sickness absence at 6 and 12 months will be compared by using multivariate logistic analysis. These analyses will be adjusted for age and sex, and duration of sick leave. The optimal cut-off value for the Keele STarT MSK and ÖMPSQ-SF tools in detecting people at high risk of prolonged sickness absence at 6 and 12 months will be determined using Receiver Operating Curve (ROC) analyses. In addition, values for sensitivity, specificity, positive and negative predictive value of RTW at 6 and 12 months will be compared using the optimal cut-off values.

A prognostic model for the risk of prolonged sickness absence, assessing risk factors listed in Table 1, will be developed according to the PROGRESS framework using multiple linear and logistic regression. The model will be externally validated in other materials in Norway, e.g. the work package 3 of the MI-NAV project and a similar project in Trondheim [31].

Multivariate logistic regression analysis will also be used to assess predictors for health care costs, and to assess if these vary across different risk profile groups.

Finally, latent class modelling will be used in order to explore if clusters of people on sick leave due to musculoskeletal disorders with regard to work-related disability and sickness absence trajectories, can be identified [32].

Separate statistical analysis plans will be developed before the data collection is finished and the database is locked. A biostatistician will contribute in the statistical analyses.

### Dissemination

The results of the study will be disseminated in several relevant research conferences. The objectives presented will form the basis of multiple articles in peer-reviewed journals and will be incorporated in the thesis of three PhD-candidates.

## Discussion

The present study aims to investigate factors that influence prolonged sickness absence, health outcomes, and costs due to a musculoskeletal disorder. We expect to identify reliable and valid tools that by themselves or in a predictive model can be used to detect people at risk of long-term sickness absence due to musculoskeletal disorders. We also expect to find predictors for high costs in people on sick leave and to present clusters of people with different work-related disabilities due to musculoskeletal disorders.

The results of this study may provide stakeholders and health care providers with tools that can be used to target high-risk individuals and may thus be important both from a socioeconomic and individual perspective. The identification of possibly modifiable risk factors may be used to targeted interventions to optimise RTW.

A possible limitation in this study may be the generalizability of the findings. A recent study showed that approximately 8% of invited patients on sick leave due to musculoskeletal disorders, accepted the invitation [33]. Given that eligible people in the current study actively must choose to participate in the study, the results may not be representative of the whole population of people on sick leave due to a musculoskeletal disorder. To reduce the selection bias, the study will include a large sample size, and people will be recruited from all over Norway.

To reduce the burden on the participants when responding to the comprehensive questionnaire, we have chosen to use single items of constructs (e.g. return-to-work expectancy and self-efficacy) instead of longer standardized questionnaires with many items. Full version questionnaires may possibly produce more informative data on some important risk factors, however, by including single-item questions we have been able to include a battery with many of the well-known risk factors.

## Data Availability

The datasets that are going to be generated and analysed during the current study will not be made publicly available due to national regulations.

## Abbreviations

COSMIN: COnsensus-based Standards for the selection of health Measurement INstruments
EQ-5D-5 L: EuroQol 5 Dimensions
ICC: Intraclass Correlation Coefficient
iPCQ: The institute of Medical Technology Assessment Productivity Cost Questionnaire
PR: Municipal Patient and User Registry
KUHR: Control and Payment of Health Refunds
MSK: Musculoskeletal
MSK-HQ: Musculoskeletal Health Questionnaire
NAV: Norwegian Labour and Welfare Administration
NPR: Norwegian Patient Registry
NSD: Norwegian Centre for Research Data
RTW: Return to Work
SDC: Smallest Detectable Change
Keele STarT MSK: Keele Subgroups for Targeted Treatment Musculoskeletal Tool
TSD: Services for Sensitive Data
ÖMPSQ-SF: Örebro Musculoskeletal Pain Screening Questionnaire short form

## References

[1] Hoy DG, Smith E, Cross M, Sanchez-Riera L, Buchbinder R, Blyth FM (2014). The global burden of musculoskeletal conditions for 2010: an overview of methods. Ann Rheum Dis.

[2] GBD 2015 Disease and Injury Incidence and Prevalence Collaborators. Global, regional, and national incidence, prevalence, and years lived with disability for 310 diseases and injuries, 1990–2015: a systematic analysis for the Global Burden of Disease Study 2015. Lancet (London, England). 2016;388(10053):1545–602. 10.1016/S0140-6736(16)31678-6 PMC5055577 27733282

[3] Woolf AD, Pfleger B (2003). Burden of major musculoskeletal conditions. Bull World Health Organ.

[4] Statistics Norway (2018). Flest til fastlegen på grunn av muskel- og skjelettlidelser.

[5] Ihlebaek C, Laerum E (2010). Hits most, costs most and gets least. TidsskrNor Laegeforen.

[6] Norwegian Labour and Welfare Administration. Sykefraværsstatistikk 2018. Available from: https://www.nav.no/no/NAV+og+samfunn/Statistikk/Sykefravar+-+statistikk/Sykefravar. Accessed 12 June 2019.

[7] Breivik H, Eisenberg E, O'Brien T (2013). The individual and societal burden of chronic pain in Europe: the case for strategic prioritisation and action to improve knowledge and availability of appropriate care. BMC Public Health.

[8] Waddell G, Burton AK (2006). Is work good for your health and well-being?.

[9] Waddell G, Burton AK (2001). Occupational health guidelines for the management of low back pain at work: evidence review. Occup Med (Oxford, England).

[10] Bendix AF, Bendix T, Haestrup C (1998). Can it be predicted which patients with chronic low back pain should be offered tertiary rehabilitation in a functional restoration program? A search for demographic, socioeconomic, and physical predictors. Spine..

[11] Hagen KB, Thune O (1998). Work incapacity from low back pain in the general population. Spine..

[12] Hedlund M, Landstad B, Wendelborg C (2007). Challenges in disability Management of Long-Term Sick Workers. Int J Disabil Manag.

[13] Cancelliere C, Donovan J, Stochkendahl MJ, Biscardi M, Ammendolia C, Myburgh C (2016). Factors affecting return to work after injury or illness: best evidence synthesis of systematic reviews. Chiroprac Manual Ther.

[14] Aasdahl L, Pape K, Vasseljen O, Johnsen R, Fimland MS (2019). Improved expectations about length of sick leave during occupational rehabilitation is associated with increased work participation. J Occup Rehabil.

[15] Hill JC, Whitehurst DG, Lewis M, Bryan S, Dunn KM, Foster NE (2011). Comparison of stratified primary care management for low back pain with current best practice (STarT Back): a randomised controlled trial. Lancet (London, England).

[16] Linton SJ, Nicholas M, MacDonald S (2011). Development of a short form of the Orebro musculoskeletal pain screening questionnaire. Spine..

[17] Hill JC, Afolabi EK, Lewis M, Dunn KM, Roddy E, van der Windt DA (2016). Does a modified STarT Back tool predict outcome with a broader group of musculoskeletal patients than back pain? A secondary analysis of cohort data. BMJ Open.

[18] Hill JC, Kang S, Benedetto E, Myers H, Blackburn S, Smith S (2016). Development and initial cohort validation of the Arthritis Research UK musculoskeletal health questionnaire (MSK-HQ) for use across musculoskeletal care pathways. BMJ Open.

[19] Beaton DE, Bombardier C, Guillemin F, Ferraz MB (2000). Guidelines for the process of cross-cultural adaptation of self-report measures. Spine..

[20] Bouwmans C, Krol M, Brouwer W, Severens JL, Koopmanschap MA, Hakkaart L (2014). IMTA Productivity Cost Questionnaire (IPCQ). Value Health.

[21] Herdman M, Gudex C, Lloyd A, Janssen M, Kind P, Parkin D (2011). Development and preliminary testing of the new five-level version of EQ-5D (EQ-5D-5L). Qual Life Res.

[22] Ilmarinen J (2007). The work ability index (WAI). Occup Med.

[23] Hill JC, Dunn KM, Lewis M, Mullis R, Main CJ, Foster NE (2008). A primary care back pain screening tool: identifying patient subgroups for initial treatment. Arthritis Rheum.

[24] Campbell P, Hill JC, Protheroe J, Afolabi EK, Lewis M, Beardmore R (2016). Keele aches and pains study protocol: validity, acceptability, and feasibility of the Keele STarT MSK tool for subgrouping musculoskeletal patients in primary care. J Pain Res.

[25] Nicolas MK, Costa DSJ, Linton SJ, Main CJ, Shaw WS, Pearce R, et al. Predicting return to work in a heterogeneous sample of recently injured workers using the brief ÖMPSQ-SF. J Occup Rehabil. 2019;29(2):295–302. 10.1007/s10926-018-9784-8 29796980

[26] Hubertsson J, Englund M, Hallgarde U, Lidwall U, Lofvendahl S, Petersson IF (2014). Sick leave patterns in common musculoskeletal disorders--a study of doctor prescribed sick leave. BMC Musculoskelet Disord.

[27] Brendbekken R, Vaktskjold A, Harris A, Tangen T (2018). Predictors of return-to-work in patients with chronic musculoskeletal pain: a randomized clinical trial. J Rehabil Med.

[28] Mokkink LB, Terwee CB, Patrick DL, Alonso J, Stratford PW, Knol DL (2010). The COSMIN study reached international consensus on taxonomy, terminology, and definitions of measurement properties for health-related patient-reported outcomes. J Clin Epidemiol.

[29] de Vet HC, Terwee CB, Mokkink LB, Knol DL (2011). Measurement in medicine.

[30] Altman DG (1991). Practical statistics for medical research.

[31] Aasdahl L, Foldal VS, Standal MI, Hagen R, Johnsen R, Solbjor M (2018). Motivational interviewing in long-term sickness absence: study protocol of a randomized controlled trial followed by qualitative and economic studies. BMC Public Health.

[32] Muthen B, Kaplan D (2004). Latent variable analysis: growth mixture modelling and related techniques for longitudinal data. Handbook of quantitative methodology for the social sciences.

[33] Aasdahl L, Pape K, Vasseljen O, Johnsen R, Gismervik S, Halsteinli V (2018). Effect of inpatient multicomponent occupational rehabilitation versus less comprehensive outpatient rehabilitation on sickness absence in persons with musculoskeletal- or mental health disorders: a randomized clinical trial. J Occup Rehabil.

